# Epidemiology of *Giardia duodenalis* assemblages in Brazil: there is still a long way to go

**DOI:** 10.1590/0074-02760200431

**Published:** 2021-01-25

**Authors:** Maria Fantinatti, Monique Gonçalves-Pinto, Luiz Antonio Pimentel Lopes-Oliveira, Alda Maria Da-Cruz

**Affiliations:** 1 Fundação Oswaldo Cruz-Fiocruz, Instituto Oswaldo Cruz, Laboratório Interdisciplinar de Pesquisas Médicas, Rio de Janeiro, RJ, Brasil; 2 Universidade do Estado do Rio de Janeiro, Faculdade de Ciências Médicas, Rio de Janeiro, RJ, Brasil

**Keywords:** Giardia duodenalis, assemblage, genotyping, human, animals, water and soil, Brazil

## Abstract

*Giardia duodenalis* infection is distributed worldwide and can achieve prevalence around 60%, especially in developing countries. This protozoan is divided into eight assemblages, in which A and B have high zoonotic potential, whereas C to H are host-specific. This scenario is changing as molecular studies progress, highlighting that knowledge on host-specificity still has a long way to go. Understanding the players involved in transmission routes enables rational designs of control strategies. Considering the high prevalence of giardiasis, this review aims to gather together the data on available studies on the distribution of *G. duodenalis* assemblages in Brazil until September 2020.

*Giardia* is an intestinal protozoan found in a wide spectrum of animal hosts (birds, reptiles and mammals). In mammals the infection occurs by *G. duodenalis*, synonymous with *G. lamblia* and *G. intestinalis*. Although there is no consensus on the most appropriate name, in this review we will use *G. duodenalis*.^1^

*G. duodenalis* present worldwide distribution, showing prevalence of 2-7% in developed countries and can reach over 30% in low and middle-income countries.^2^ High frequencies are observed in poorer countries as they are closely associated with poor sanitation infrastructure. Although the water supply system is the main source of *G. duodenalis* transmission in outbreaks, in endemic areas direct transmission has great epidemiological relevance.

The great genomic diversity found in the *G. duodenalis* species meant that its subspecies were initially organised into groups, which are currently called assemblages or genotypes. The first divisions in *G. duodenalis* assemblages were carried out according to specificity by the host from which the isolate originated.^3^ ^,^ ^4^ ^,^ ^5^ This subdivision was corroborated in intrinsic characteristics of the parasite, such as antigenic factors and isoenzymes, but mainly by DNA analysis, which confirmed the heterogeneity of *G. duodenalis*.^4^ ^,^ ^5^

Nowadays, the *G. duodenalis* species is phylogenetically divided into eight assemblages, classified from A to H.^4^ ^,^ ^6^ Classically, assemblages A and B are considered potentially zoonotic, as they infect humans and a wide variety of mammals, while assemblages C-H are considered host-specific. Assemblages C and D are associated with infection in dogs, assemblage F in felines, assemblage G in rodents and assemblage H in marine mammals.^5^ ^,^ ^7^ The assemblage E was considered only infecting ungulate and biungulate animals, but the human infection has been reported.^8^ ^,^ ^9^ ^,^ ^10^ Yet, the affinity of these assemblages for the hosts has been constantly discussed and updated.^11^

When symptomatic, the most frequent symptoms of giardiasis are consequence of acute or chronic diarrhoea, such as abdominal colic, flatulence, dehydration, nausea, vomiting and fatigue.^6^ ^,^ ^12^ There is no proven association between clinical manifestations and the infecting assemblage. Symptomatic and asymptomatic cases were already observed for all assemblages, especially in humans.^13^ However, studies correlating specific symptoms, as well as disease severity, differ in its results. Some authors report assemblage B as more associated with clinical manifestations,^14^ ^,^ ^15^ ^,^ ^16^ ^,^ ^17^ while others find the same association with assemblage A.^18^ ^,^ ^19^ ^,^ ^20^ Similarly, no relationship between the immune response profile and a specific infecting assemblage was observed, although assemblages A, B, and E are associated with damage to the intestinal mucosa in humans.^21^ ^,^ ^22^

The different molecular characterisation tools have played an important role not only to better understand the characteristics of the assemblages, but also to understand the zoonotic cycles of transmission. Further genotyping studies are needed in order to elucidate the complexity involved in the transmission dynamics of *G. duodenalis* assemblages, especially in regions with high frequencies of infection.

Brazilian studies about the epidemiology of giardiasis indicates that the *G. duodenalis* frequency in children can be higher than 60%.^23^ ^,^ ^24^ ^,^ ^25^ ^,^ ^26^ ^,^ ^27^ The numbers demonstrate the need for control strategies for this infection. However, in view of the low perspective of implementing the universal sanitation, it is essential to know the sources of contamination and the possible transmission routes in order to search strategies of prophylaxis. Thus, understanding the epidemiology of *G. duodenalis* assemblages could contribute to reducing giardiasis. In this context, this review aimed to describe the distribution of *G. duodenalis* assemblages in Brazil between 2007-2020.

## MATERIALS AND METHODS

A review was carried out based on an exploratory and descriptive bibliographic survey in September, 2020, with the main theme: “*Giardia duodenalis* assemblages circulating in Brazil”. The electronic database, Medical Literature Analysis and Retrieval System on-line (Medline), was used.

**Fig. 1:**
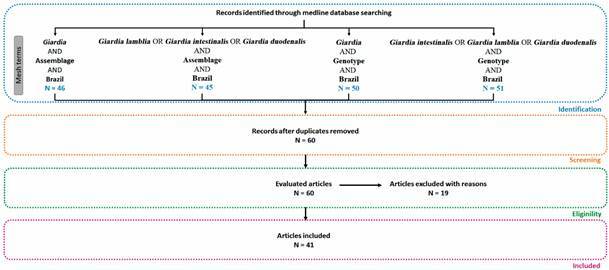
methodological search strategy for Medline bibliographic survey on *Giardia duodenalis* assemblages in Brazil.

Medical subject headings (MeSH) terms were used to define the search for: (1) etiological agent: “Giardia”; (2) characteristic of the etiological agent: “assemblage” or “genotype”; (3) geographical area: “Brazil”. To search with the aforementioned descriptors, the Boolean operator “and” was used. Thus, we obtained the following research strategy: (1) [(Giardia) AND assemblage] AND Brazil; (2) [(*Giardia lamblia*) OR (*Giardia intestinalis*) OR (*Giardia duodenalis*) AND assemblage] AND Brazil; (3) [(Giardia) AND (genotype)] AND Brazil; (4) [(*Giardia intestinalis*) OR (*Giardia lamblia*) OR (*Giardia duodenalis*) AND (genotype)] AND Brazil (Fig. 1).

The following inclusion criteria were used for the selection of manuscripts: articles retrieved in full, published in English until the year 2020. The following were excluded: studies that did not perform genotyping of the *Giardia* isolates (n = 5); studies that performed analysis *in silico* or from data available from databases (n = 2); studies that were not performed with isolates from Brazil (n = 2); *in vitro* and experimental model studies that did not perform genotyping of original isolates (n = 4); studies that used DNA from single cysts of *G. duodenalis* separated using a micromanipulation technique (n = 1); studies on archaeological material (n = 2); studies that did not use original *G. duodenalis* genotyping results and that had already been addressed in previous papers (n = 2); studies review (n = 1).

## RESULTS AND DISCUSSION

Frequency and distribution of genotyping studies in Brazil

Knowledge of the geographic distribution of parasitosis, such as giardiasis, is essential to target specific control measures. In Brazil, giardiasis is a public health problem with an unknown prevalence. Most of the studies in this field results from research by individual groups, and are usually sectional and aimed at local scope parasitological surveys. The lack of information about the transmission dynamics of *G. duodenalis* in the country makes it difficult to implement strategic control actions that are targeted to potential sources of transmission. The scarcity of studies associated with the absence of a national infection survey makes it hard to understand the evolution of parameters related to the frequency of giardiasis in the population. Currently, it is not possible to say whether the *G. duodenalis* distribution in Brazil is changing upwards or downwards.

**Fig. 2:**
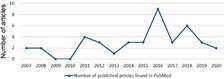
articles published on Medline about *Giardia* assemblage from Brazil. The points represent the number of published articles found in the search.

Although there are many areas with high frequencies of *G. duodenalis* infection (over 40%), all genotyping studies are relatively recent, with the first one dating back to 2007.^12^ We have observed a modest progress in these studies but with a low annual frequency (Fig. 2). In the period 2007-2020, only 41 studies were identified on Medline (Table I). This search tool has limitations as journals fulfilling technical criteria to be available in the Medline research base, are considered with the predetermined MeSH terms. Then, an underestimated number of studies published in the grey literature was not considered herein. However, the strategy used ensures reproducibility and guarantees a sample of studies carried out in Brazil.

Brazil is a country of large continental proportions (8,516,000 km²), composed of 27 states and a federal district divided into five regions (North, Northeast, Midwest, Southeast and South). Of the Brazilian regions, one of them (Midwest) does not present any studies of *G. duodenalis* genotyping, while two others (North and Northeast) share only two studies, each carried out from human samples. Most of the studies were developed in the Southeast region (30/41). This region is responsible for the main share of the country’s gross domestic product (GDP), and despite its small geographic dimension, it concentrates a large number of research institutes and postgraduate courses (48.45%).^29^

Brazil has the greatest biodiversity on the planet. It is estimated that about 11,000 known vertebrate animal species and 210 species of mammals have already been observed.^30^ However, little research has been performed on *Giardia* and its genotypes in wild animal studies. All studies were carried out using samples of wild animals that had some proximity to humans (captive animals or animals in parks) which would facilitate transmission.^31^ ^,^ ^32^ ^,^ ^33^ There are no studies on the circulation of *G. duodenalis* assemblages in these animals in a natural environment.

Most studies (34/41) of *G. duodenalis* genotyping are carried out with samples of humans, domestic animals or farm animals (Table I). Up to now, it is not possible to establish a relationship between the *G. duodenalis* assemblage and the pathogenesis of giardiasis. Infections in humans have been further explored due to its importance in public health. The approach of man to pets increases the importance of research on transmission routes, including research by this group. Although farm animals have less affective proximity to humans, infection by *G. duodenalis* can impact the weight gain of these animals. Then, studies with these hosts are also relevant for the economy.^34^ ^,^ ^35^

Despite the high prevalence of *Giardia* in humans, and the largest diversity of animals in the world, the Brazilian contribution to the understanding of *G. duodenalis* diversity is scarce. In relation to developed countries, such as Portugal, Spain, Italy and China, Brazil has few studies of *G. duodenalis* genotyping, and most of them are concentrated in a single region. Further studies on the topic should be promoted, both in humans and in domestic, farm and wild animals, in order to assist in understanding the dynamics of transmission of *G. duodenalis*.

**TABLE I:** Genotyping studies of *Giardia duodenalis* in Brazil up to September 2020

Year	Author	State	Isolate origin	Genotyping tool	Assemblage found				
					*βg*	*gdh*	*tpi*	*SSU-rRNA*	*orfC4*
2020	Pineda et al.	São Paulo	Pool water	Sequencing	A	NI	NI	NI	
	Corrêa et al.	São Paulo	Human	Sequencing	A, B	A, B	A, B		
2019	de Aquino et al.	São Paulo	Buffalo	Sequencing	E	E	E	E	
	Yamashiro et al.	São Paulo	Water	Sequencing	NI	NI	A	NI	
			Sewage		B		C		
	Pacheco et al.	Bahia	Human	PCR-RFLP	A, B	A, B			
				Sequencing	A, B				
2018	Fantinatti et al.	Rio de Janeiro	Dog	Sequencing	A	A			
	Ferreira et al.	Paraná	Vegetable	Sequencing		E	NI	NI	
			Soil	Sequencing		E	NI	NI	
			Water	Sequencing		E	NI	NI	
	Seguí et al.	Paraná	Human	Sequencing	A	A, B			
	De Araújo et al.	São Paulo	Surface raw water	qPCR (taqman)		A			
	Leal et al.	São Paulo	Oyster	Sequencing			A		
			Estuarine water	Sequencing			A, C		
	Nunes et al.	Amazonas	Human	Sequencing	A, B	A, B	A, B		
		Ceará	Human	Sequencing	A, B	NI	NI		
		Piauí	Human	Sequencing	A, B	A, B	A, B		
2017	Rafael et al.	Paraná	Vegetable	PCR-RFLP		A, B, E			
	Faria et al.	Rio de Janeiro	Human	Sequencing	A, B	A, B	A, B		
	Da Cunha et al.	Minas Gerais	Toco toucan	Sequencing				A	
2016	Ulloa-Stanojlović et al.	São Paulo	Wastewater	PCR (assemblage-specific)		A, B			
	Fantinatti et al.	Rio de Janeiro	Human	Sequencing	A, E	A, E			
	Quadros et al.	Santa Catarina	Human	PCR-RFLP		A, B			
			Dog	PCR-RFLP		A, B, C			
	Faria et al.	Rio de Janeiro	Human	PCR-RFLP	A, B	A, B			
				qPCR (assemblage-specific)		A, B	A, B		A, B
	Coronato Nunes et al.	Amazonas	Human	Sequencing	A, B				
	Scalia et al.	Minas Gerais	Human	Sequencing	A, B, E	A, B	A, B		
	Tiyo et al.	Paraná	Vegetable	Sequencing		A			
	Fava et al.	Minas Gerais	Dog	Sequencing		D	A, B, C, E		
	Oliveira-Arbex	São Paulo	Human	Sequencing	A, B	A, B	A, B		
2015	Colli et al.	Paraná	Human	PCR-RFLP	A, B	A, B			
				Sequencing		A, B			
	David et al	São Paulo	Human	Sequencing	A, B	A, B	A, B		
			Dog	Sequencing	A, C, D	A, C, D	A, C		
	Colli et al.	Paraná	Human	PCR-RFLP	A, B	A, B			
				Sequencing		A, B			
			Dog	PCR-RFLP	NI	NI			
				Sequencing		B, C, D			
			Vegetable	PCR-RFLP	NI	NI			
				Sequencing		B			
2014	Durigan et al.	São Paulo	Human	Sequencing	A, B	A, B	A, B, C		
			Cat	Sequencing		D	A,B		
				PCR (assemblage-specific)			D		
			Dog	Sequencing	D	D	A, B, C		
				PCR (assemblage-specific)			C, D		
			Cattle	Sequencing	E	E	A		
			Water	Sequencing	NI	A,B,D	B,C		
	Paz e Silva et al.	São Paulo	Sheep	PCR-RFLP		E			
				Sequencing		E			
	David et al.	São Paulo	Nonhuman primates	Sequencing		A	A		
2013	Fava et al.	Minas Gerais	Cattle	Sequencing		E	A, B, E		
			Pig	Sequencing		E	NI		
			Sheep	Sequencing		E	E, B		
2012	Santos et al.	Minas Gerais	Human	Sequencing		B			
	Paz e Silva et al.	São Paulo	Cattle	Sequencing		A, E		A, E	
				PCR-RFLP		A, E		-	
	Paz e Silva et al.	São Paulo	Dog	Sequencing	C, D	C, D		-	
				PCR-RFLP	C, D	C, D		-	
2011	Fernandes et al.	São Paulo	Water	Sequencing		A			
			Sewage	Sequencing		A, B			
	Volotão et al.	São Paulo	Human	Sequencing	A				
			Dog	Sequencing	A				
	Gomes et al.	Minas Gerais	Human	PCR (assemblage-specific)				A	
			Dog					A	
	Soares et al.	Uninformed	Jaguar	Sequencing		A		A	
			Nonhuman primates	Sequencing		B		B	
			Chinchilla	Sequencing		B		B	
			Ostriches	Sequencing		B		B	
2008	Volotão et al.	Santa Catarina	Nonhuman primates	Sequencing	A				
	Kohli et al.	Ceará	Human	qPCR multiplex				A, B	
2007	Souza et al.	São Paulo	Human	Sequencing		A, B			
			Cat	Sequencing		A, F			
			Dog	Sequencing		C, D			
			Cattle	Sequencing		A, E			
	Volotão et al	Rio de Janeiro	Human	Sequencing	A				
				PCR-RFLP	A				
			Dog	Sequencing	A				
				PCR-RFLP	A				
			Cat	Sequencing	A				
				PCR-RFLP	A				

The studies were grouped by year of publication, authors, state where the study was carried out, the isolate origin, the tool for genotyping used and the assemblages found according to the gene target used. The hatched area represents a genetic target not used in the methodological strategy. NI: assemblage not identified by the study; hatch: gene targets not used in the methodological strategy; PCR-RFLP: polymerase chain reaction restriction fragment length polymorphism; qPCR: quantitative polymerase chain reaction.

**Genotyping strategies of *G. duodenalis* in Brazil**

Many genes have been proposed, and 16 targets for molecular characterisation are described, divided according to the potential to discriminate species of the genus *Giardia* and/or discriminate *G. duodenalis* assemblages.^36^ However, due to the high frequency of single nucleotide polymorphisms, there are few genes used in the literature for genotyping. Initially, the most used gene was small subunit ribosomal ribonucleic acid (SSU rRNA), as it is extremely conserved;^37^ however, the amplification of the locus by polymerase chain reaction (PCR) can present difficulties.^6^ Thus, for *G. duodenalis* typing, the genes most widely used are those that encode the proteins Triose Phosphate Isomerase (*tpi*),^38^ Glutamate Dehydrogenase (*gdh*)^3^ and Beta Giardin (βg)^39^. In Brazil, 83% of the *G. duodenalis* genotyping studies carried out from 2007-2019 (29/35) used the sequencing of regions of classically-used genes. β*g*, *gdh* and/or *tpi* were used in all studies, with the exception of one research study that used only the target SSU-rRNA.^33^ Six studies that used the SSU-rRNA marker in a multiplex approach with *bg*, *gdh* or *tpi* (Table I).^32^ ^,^ ^40^ ^,^ ^41^ ^,^ ^59^ ^,^ ^60^ ^,^ ^61^

The low investment in tools with discriminatory potential results that sequencing is still the most widely used methodology, although it may present divergences according to the gene target used. The whole genome sequencing (WGS) is suggested for genotyping;^10^ however, the high cost and the delay in obtaining results prevent its use on a large scale, especially in developing countries, such as Brazil.

In Brazil, assemblage-specific PCR was used in four studies, mainly with the *gdh* and *tpi* markers, but *orfC4* and *SSu-rRNA* were also observed.^42^ ^,^ ^43^ ^,^ ^44^ ^,^ ^45^ ^,^ ^46^ PCR or quantitative PCR (qPCR) were used to identify assemblages A and B in isolates from human clinical samples, surface raw water or wastewater.^43^ ^,^ ^44^ ^,^ ^45^ These results were identical to those of PCR restriction fragment length polymorphism (PCR-RFLP), and no divergence or inconsistencies in the assemblages were found among the four different loci (qPCR: *gdh*, *tpi*, *orfC4*; PCR-RFLP: *βg*, *gdh*).^43^ In isolates from dog samples, some divergences between assemblage-specific PCR were found for C and D, using the *tpi* target, when compared to gene sequencing.

PCR-RFLP was used for genotyping in ten studies, most of which were accompanied mainly by gene sequencing (8/10).^21^ ^,^ ^28^ ^,^ ^40^ ^,^ ^43^ ^,^ ^47^ ^,^ ^48^ ^,^ ^49^ ^,^ ^50^ ^,^ ^51^ ^,^ ^52^ The most used target in PCR-RFLP was *gdh*, and sometimes appeared combined with the target *βg*.^21^ ^,^ ^43^ ^,^ ^47^ ^,^ ^48^ ^,^ ^49^ ^,^ ^50^ ^,^ ^51^ ^,^ ^52^ Only one study used only the *βg* target for genotyping in PCR-RFLP.^28^ In these studies, PCR-RFLP showed a satisfactory result in identifying the assemblages circulating, with the exception of just one study where PCR-RFLP data obtained by sequencing did not determine the assemblage of isolates from samples of humans, dogs and vegetables.^49^

The relevance of the whole genetic sequence for a better understanding of *G. duodenalis* isolates is undeniable; however, all other sequencing brings an extremely relevant contribution to the scientific literature. The choice of the genotyping tool must be determined according to the investigation focus. PCR-RFLP proved to be a good strategy for an initial survey of circulating assemblages. Assemblage-specific PCR can be of great value in places where infectious assemblages are already known. Due to the divergences between the gene targets used in the sequencing, the use of more than one marker is recommended, as data from multi-locus sequencing brings more robustness to understanding the assemblage transmission dynamics. However, new gene markers and new financially viable tools are urgently needed to stimulate the expansion of genotyping studies and, consequently, enable a better understanding of the real zoonotic potential of *G. duodenalis* assemblages.

**Circulation of *G. duodenalis* assemblages in environmental samples from Brazil**

The presence of intestinal parasites dates from antiquity. In Brazil, the findings in coprolites and mummies point to the existence of these infections for more than 7,000 years and the existence of *Giardia*, for more than 5,300 years.^53^ However, isolating cysts from archaeological material is an essential but difficult task, and techniques continue to be improved.^54^ ^,^ ^55^ Thus, further studies are needed to determine the genetic characteristics of the *G. duodenalis* isolates circulating at that time.

The occurrence of *Giardia* in water and food is associated with contamination by faeces from humans and/or animals, so different species and assemblages can be found. The main form of *G. duodenalis* infection, reported mainly in cases of outbreaks, is via waterborne transmission.^56^ Thus, assessing the quality of drinking water sources is an excellent measure of giardiasis control, and genotyping the water isolates and the individuals who consume it helps to outline the transmission cycles.

**Fig. 3:**
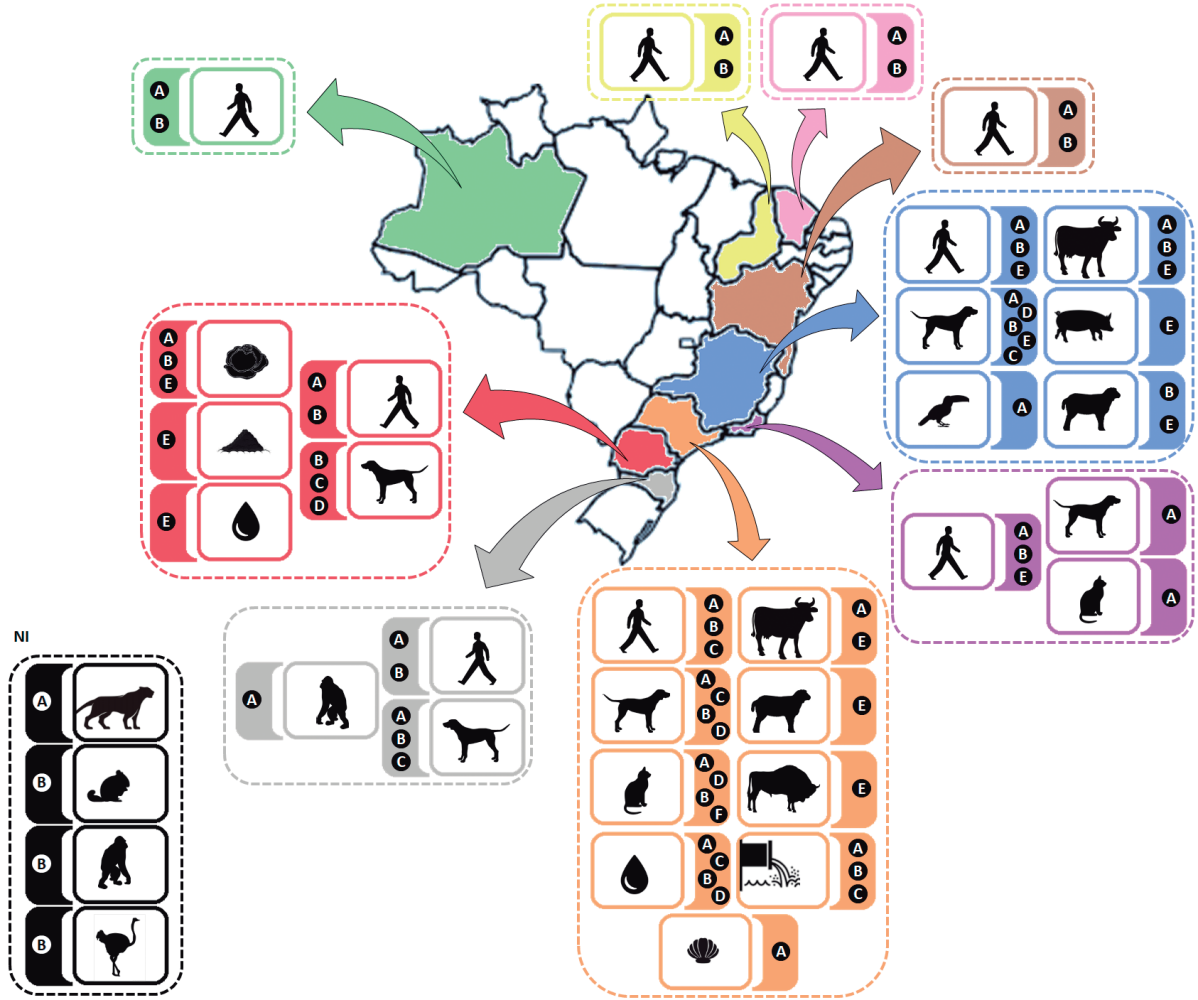
distribution of *Giardia duodenalis* assemblage in Brazil. The states where *G. duodenalis* genotyping were not found are presented whithout colour. The arrows indicate the hosts and the respective assemblages observed.

In Brazil, genotyping studies of *G. duodenalis* in water are basically concentrated in the State of São Paulo,^42^ ^,^ ^44^ ^,^ ^45^ ^,^ ^57^ ^,^ ^58^ ^,^ ^59^ ^,^ ^61^ and in the State of Paraná, which presents a single study^41^ (Table II) (Fig. 3). The presence of *G. duodenalis* assemblages A, B, C, D and E suggests the contamination of water sources by faeces from different groups of hosts.

**TABLE II:** *Giardia duodenalis* assemblages found in animals and in the environment in Brazilian states

State	Isolate origin	Assemblage
Amazonas	Human	A^72^ ^,^ ^76^
		B^72^ ^,^ ^76^
Ceará	Human	A^68^ ^,^ ^76^
		B^68^ ^,^ ^76^
Piauí	Human	A^76^
		B^76^
Bahia	Human	A^21^ B^21^
Minas Gerais	Human	A^46^ ^,^ ^74^
		B^70^ ^,^ ^74^
		E^74^
	Dog	A^46^ ^,^ ^84^
		B^84^
		C^84^
		D^84^
		E^84^
	Cattle	A^86^
		B^86^
		E^86^
	Pig	E^86^
	Sheep	B^86^
		E^86^
	Toco toucan	A^33^
Rio de Janeiro	Human	A^8^ ^,^ ^28^ ^,^ ^43^ ^,^ ^75^
		B^43^ ^,^ ^75^
		E^8^
	Dog	A^28^ ^,^ ^83^
	Cat	A^28^
São Paulo	Human	A^42^ ^,^ ^67^ ^,^ ^69^ ^,^ ^71^ ^,^ ^73^ ^,^ ^78^
		B^42^ ^,^ ^67^ ^,^ ^71^ ^,^ ^73^ ^,^ ^78^
		C^42^
	Dog	A^42^ ^,^ ^69^ ^,^ ^71^
		B^42^
		C^42^ ^,^ ^47^ ^,^ ^67^ ^,^ ^71^
		D^42^ ^,^ ^47^ ^,^ ^67^ ^,^ ^71^
	Cat	A^42^ ^,^ ^67^
		B^42^
		D^42^
		F^67^
	Buffalo	E^60^
	Cattle	A^40^ ^,^ ^42^ ^,^ ^67^
		E^40^ ^,^ ^42^ ^,^ ^67^
	Sheep	E^50^
	Nonhuman primates	A^85^
	Oyster	A^58^
	Water and/or Sewage	A^44^ ^,^ ^45^ ^,^ ^57^ ^,^ ^58^ ^,^ ^59^ ^,^ ^61^
		B^57^ ^,^ ^59^ ^,^ ^42^ ^,^ ^44^
		C^58^ ^,^ ^59^
		D^42^
Paraná	Human	A^48^ ^,^ ^49^ ^,^ ^77^
		B^48^ ^,^ ^49^ ^,^ ^77^
	Dog	B^49^
		C^49^
		D^49^
	Vegetable	A^51^ ^,^ ^62^
		B^49^ ^,^ ^51^
		E^41^ ^,^ ^51^
	Water	E^41^
	Soil	E^41^
Santa Catarina	Human	A^52^
		B^52^
	Dog	A^52^
		B^52^
		C^52^
	Nonhuman primates	A^31^
Uninformed	Jaguar	A^32^
	Monkey	B^32^
	Chinchilla	B^32^
	Ostriches	B^32^

The two studies that genotyped *G. duodenalis* isolates from sewage samples identified the presence of assemblages A, B and C (Table I).^57^ ^,^ ^59^ More studies like this should be encouraged, because quantifying the presence of intestinal parasites in sewage could be a strategy to predict the contamination index of the human and animal population by agents such as *G. duodenalis*.

In addition to waterborne transmission, giardiasis is also a foodborne disease. In Brazil, genotyping studies of *G. duodenalis* from food have only been observed in the State of Paraná (Tables I-II) (Fig. 3).^41^ ^,^ ^49^ ^,^ ^51^ ^,^ ^62^ The presence of assemblages A, B and E on the surface of vegetables was also investigated. It is common to use horse and cattle faeces as vegetable fertilisation strategies, and this could justify the presence of isolates of assemblage E in these foods. However, it is worth mentioning that the plants undergo a constant irrigation process and that the assemblage (E) has already been observed in water source.^41^ As assemblages A and B can also be found in these vegetables,^33^ ^,^ ^35^ ^,^ ^40^ the interpretation can be equivalent. However, in Brazil, the cultivation of vegetables is still predominately artisanal and familiar, although carried out in large fields, which results in human contact and contact with other animals that have been raised on the land.

***Giardia duodenalis* assemblage’ circulation in host from Brazil**

Classically, *G. psittaci* and *G. ardeae* species are commonly reported in bird droppings and *G. duodenalis* in mammal infections.^5^ ^,^ ^6^ However, recent reports have demonstrated the circulation of the following *G. duodenalis* assemblages in bird species: A, B, D and F from Spain,^63^ ^,^ ^64^ A from Italy,^65^ and A and B from the Ivory Coast.^66^ In Brazil, the first report of this parasite in birds occurred in toco toucan which was infected by assemblage A.^33^ As this is a bird that lives in captivity, the infection may have occurred through contact with human faeces, which is corroborated by the finding of this assemblage (A) (Tables I-II) (Fig. 3). The possibility of *G. duodenalis* infection in birds could greatly increase its potential for dispersion.

Although the division of *G. duodenalis* assemblage by association with the host it infects is still maintained today, the findings indicate that its current distribution may be much more complex. Many doubts persist when we observe species infected by assemblages outside the expected host-specific species: (1) Is it possible that the circulation of these assemblages has always occurred and the scarcity of studies has hindered their previous identification? (2) Is it possible that the high frequency of interspecific contact could increase the pressure of infection and consequently select individuals capable of infecting this new host?

In Brazil, assemblages A, B, C and E have already been identified in isolates from human faeces samples distributed in 21 studies carried out in the nine states.^8^ ^,^ ^12^ ^,^ ^42^ ^,^ ^43^ ^,^ ^46^ ^,^ ^48^ ^,^ ^49^ ^,^ ^52^ ^,^ ^59^ ^,^ ^67^ ^,^ ^68^ ^,^ ^69^ ^,^ ^70^ ^,^ ^71^ ^,^ ^72^ ^,^ ^73^ ^,^ ^74^ ^,^ ^75^ ^,^ ^76^ ^,^ ^77^ ^,^ ^78^ Assemblages A and B are classically associated with infection in humans (Table I) (Fig. 3).^5^ ^,^ ^6^ However, the identification of assemblages C and E points to the possibility of man’s participation in the transmission cycles of these assemblages. Assemblage E was reported in humans by our group in Rio de Janeiro,^8^ and also by researchers from Minas Gerais.^74^ However, assemblage C has only been reported in São Paulo.^42^ Other authors have reported the occurrence of assemblages C^79^ ^,^ ^80^ ^,^ ^81^ and E^9^ ^,^ ^82^ in humans from other countries. Of the assemblages already identified in humans, only assemblage F was not yet identified in Brazil.^11^ Possibly, the low frequency of this assemblage, even among felines in the country, has not favoured the occurrence of human infection.

Domestic animals have great relevance in *G. duodenalis* zoonotic transmission, mainly anthropozoonotic. The two genotyping studies of *G. duodenalis* in cats carried out in São Paulo^42^ ^,^ ^67^ and one in Rio de Janeiro (one animal),^28^ point to the circulation of assemblage A (Table II) (Fig. 3). In Brazil, the feline host-specific assemblage (F) was only observed in São Paulo,^67^ where the circulation of assemblages B and D was also observed.^42^

In dogs, infection by host-specific assemblages (C and D) was observed in Minas Gerais, São Paulo, Santa Catarina and Paraná (Table II) (Fig. 3).^42^ ^,^ ^47^ ^,^ ^49^ ^,^ ^52^ ^,^ ^67^ ^,^ ^71^ ^,^ ^84^ The infection by assemblage E, which is host-specific for farm animals, was reported in Minas Gerais.^83^ Although they used multilocus genotyping, some studies observed a large divergence of the data obtained from the two markers used (*tpi* and *gdh*). This could explain the identification of many different assemblages in these dog samples^42^ ^,^ ^84^ (Table I).

In Rio de Janeiro, only assemblage A was identified in dogs.^28^ ^,^ ^83^ However, dogs infected by this assemblage (A) were also observed in other states: São Paulo,^42^ ^,^ ^69^ ^,^ ^71^ Minas Gerais,^46^ ^,^ ^84^ Santa Catarina.^52^ Assemblage B was observed in Minas Gerais,^84^ Santa Catarina^52^, São Paulo^42^ and Paraná^49^ (Table II) (Fig. 3)

In Brazil, all assemblages (A, B, C, D, E and F) were identified in domestic animals, demonstrating the epidemiological importance of pets in maintaining cysts in the environment. The identification of assemblages A or B circulating in pets suggests that hosts other than dogs and cats may be involved in the transmission cycles. However, the genotyping of *G. duodenalis* isolates from these domestic animals still remains an unknown field.

Even considering the scarce knowledge of *G. duodenalis* in infecting wild animals, assemblages with high anthropozoonotic potential (A and B) were already identified in these animals (non-human primates, ostriches, chinchillas, jaguar, toucan).^31^ ^,^ ^32^ ^,^ ^33^ ^,^ ^85^ All animals surveyed had some proximity to humans, which could justify the circulation of these assemblages.

In farm animals (cattle, sheep, pig, and buffalo), the assemblages A, B and E were reported, as expected (Tables I-II) (Fig. 3).^40^ ^,^ ^42^ ^,^ ^50^ ^,^ ^60^ ^,^ ^67^ ^,^ ^86^ In these animals, the identification of assemblages A and B, as well as assemblage E, highlights the possible participation of humans, and even domestic animals such as dogs, in the transmission cycles.

In Brazil, assemblages G and H were not yet identified, probably due to the scarcity of studies in rodents and marine mammals. From marine waters, only isolates found in oysters were phylogenetically classified as assemblage A.^58^ As these molluscs function as filtering organisms, it cannot be ruled out that assemblage A comes from contaminated environments. It is worth noting that pseudoparasitism cannot be excluded as a possibility in isolated cases of unexpected assemblages in certain hosts.

Final considerations

Brazil is a country with extensive territorial proportions, which means that it presents geographical, climatical biomes with cultural and GDP differences. However, there is a lack of knowledge about the *G. duodenalis* assemblages circulating in the country, and most of the regions still need to be explored. *G. duodenalis* genotyping is an important strategy to learn about the genetic variability of the species, mainly, to help understand potential transmission cycles. Despite the high frequency of this protozoa, especially in Brazilian children, little has been explored about the epidemiology of assemblages in humans and animals. In wild animals, we do not know whether *G. duodenalis* infection occurs in the natural environment and which circulating assemblages would be involved in this case. By sequencing or PCR strategies, and its variations, the circulation of assemblages A, B, C, D, E and F are being reported. Assemblage A in particular, and also assemblage B, have a high anthropozoonotic potential, so their occurrence is expected in different host species. The others assemblages (C-H) are considered host-specific, but can cause the infection of different, atypical hosts, as observed in human infection by assemblage C (host-specific for dogs) and E (considered host-specific for farm animals), in canine infection by assemblage E and in toucan infection by assemblage A. Despite these data, given that few areas of the country were studied and also that few hosts were investigated, knowledge about the real epidemiology of *G. duodenalis* assemblages in Brazil is still a long way to go.
